# Pharmacological therapies for children with type 2 diabetes mellitus should be individualized

**DOI:** 10.1186/1687-9856-2013-S1-P32

**Published:** 2013-10-03

**Authors:** T Urakami, M Okuno, A Yoshida, J Suzuki, H Mugisima

**Affiliations:** 1 Department of Pediatrics, Nihon University School of Medicine, Tokyo, Japan

## Aim

We presently prescribe a variety of pharmacologic therapies tailored to each patient’s characteristics. We studied the current pharmacologic therapies at our outpatient clinic in obese and non-obese children with type 2 diabetes mellitus (T2DM).

## Methods

We treated 108, 80 obese and 28 non-obese, children diagnosed as having T2DM. Among these patients, 26 obese and 23 non-obese children were assigned to pharmacological therapies during the course of diabetes. The frequency of progression to pharmacologic therapies was significantly higher in non-obese than in obese patients (82.1% vs. 32.5%, P<0.05). As to the indication for pharmacologic therapies, oral hypoglycemic drugs (OHD) and/or insulin were started if the HbA1c value exceeded 7.0% despite dietary and exercise management.

## Results

1) For the 27 obese patients, metformin alone or in combination with an additional medication was frequently used, i.e. 6 patients received metformin alone and 9 metformin with additional OHD including an α-glucosidase inhibitor and/or thiazolidinedione. Only 2 patients independently received glimepiride as a sulfonylurea (SU). In addition, 9 patients were treated with insulin, using basal insulin supported with OHD or biphagic pre-mixture insulin. 2) On the other hand, the 23 non-obese patients were frequently treated with insulin alone or in combination with an additional medication followed by SU, i.e. 11 patients received insulin alone or with additional OHD, and 9 used glimepiride alone or in combination with other OHD. 3) New anti-diabetic drugs, a DPP-4 inhibitor and a GLP-1 receptor agonist, seemed to exert positive effects on glycemic control without occurrence of hypoglycemic episodes in some patients regardless of the type of diabetes. The non-obese patients tended to require pharmacologic therapy, in particular insulin, at an earlier stage of diabetes as compared to the obese patients. 4) Some patients using insulin experienced mild hypoglycemia, but no other significant adverse events were observed with any of the medications.

## Conclusion

We identified differences among pharmacologic therapies between obese and non-obese children with T2DM. New anti-diabetic drugs, an incretin mimetic and an enhancer, seemed to be effective for some young patients, showing efficacy similar to that in adult T2DM patients. These results suggest that pharmacologic treatment strategies in childhood T2DM should be tailored to individual patient characteristics.

